# Monte Carlo Simulation of Pesticide Toxicity for Rainbow Trout (*Oncorhynchus mykiss*) Using New Criteria of Predictive Potential

**DOI:** 10.3390/jox15030082

**Published:** 2025-06-01

**Authors:** Alla P. Toropova, Andrey A. Toropov, Emilio Benfenati

**Affiliations:** Istituto di Ricerche Farmacologiche Mario Negri IRCCS, Department of Environmental Health Science, Via Mario Negri 2, 20156 Milan, Italy; andrey.toropov@marionegri.it (A.A.T.); emilio.benfenati@marionegri.it (E.B.)

**Keywords:** CORAL software, Monte Carlo method, pesticide toxicity, QSAR, rainbow trout

## Abstract

*Background*: The toxicity of pesticides for fish in general and Rainbow Trout (*Oncorhynchus mykiss*) in particular is an important ecological indicator required by regulations, and it implies the use of a large number of fish. The number of animals needed would be even higher to evaluate metabolites and pesticide impurities. Considering ethical issues, the costs, and the necessary resources, the use of in silico models is often proposed. *Aim of the study*: We explore the use of advanced Monte Carlo methods to obtain improved results for models testing Rainbow Trout (*Oncorhynchus mykiss*) acute toxicity. Several versions of the stochastic Monte Carlo simulation of pesticide toxicity for Rainbow Trout, carried out using CORAL software, were studied. The set of substances was split into four subsets: active training, passive training, calibration, and validation. Modeling was repeated five times to enable better statistical evaluation. To improve the predictive potential of models, the index of ideality of correlation (IIC), correlation intensity index (CII), and coefficient of conformism of correlation prediction (CCCP) were applied. *Main results and novelty*: The most suitable results were observed in the case of the CCCP-based optimization for SMILES-based descriptors, achieving an R^2^ of 0.88 on the validation set, in all five random splits, demonstrating consistent and robust modeling performance. The relationship of information systems related to QSAR simulation and new ideas is discussed, assigning a key role to fundamental concepts like mass and energy. The study of the mentioned criteria of predictive potential during the conducted computer experiments showed that even though they are all aimed at improving the predictive potential, their values do not correlate, except for the CII and the CCCP. This means that, in general, the information impact of the considered criteria has a different nature, at least in the case of the simulation of toxicity for Rainbow Trout (*Oncorhynchus mykiss*). The applicability domain of the model is specific for pesticides; the software identifies potential outliers by looking at rare molecular fragments.

## 1. Introduction

Modern agriculture is a multifunctional system of supplying consumers with numerous needs, from citrus fruits [1,2,3] to seafood [4,5,6,7,8,9,10]. Chemical pollution caused by the agricultural industry significantly impacts customers’ health. Hence, technologies that are able to at least reduce the harm of dangerous chemical pollutants are necessary. For instance, owing to the valorization of agro-wastes, citrus by-products can be a precious source of energy and natural beneficial compounds used for manifold applications from biofuels to dietary supplements [11]. A group of toxins produced by Alternaria fungi frequently contaminate tomatoes and tomato products [12], which should be controlled using corresponding fungicides. In relation to seafood, it becomes really necessary to maintain the bio-economy of proteins used in food and medicine applications [13].

Chemical pollution caused by the agricultural industry significantly impacts customers’ health. Hence, technologies that are able to at least reduce the harm of dangerous chemical pollutants are necessary. In general, pesticides are widely used in agricultural activities, and there is a debate because they, while improving the economic performance of agricultural processes, nevertheless pollute the environment. The continuing growth of chemical pollution requires a comprehensive assessment of the hazard and risk of pesticides used to increase crop yields; indeed, pesticides may severely affect aquatic and other ecosystems [9,10,14,15]. In relation to aquatic environments, toxicity to fish is perhaps the most informative and used environmental indicator. A reliable assessment of toxicity to fish and the effects of substances that are pesticides, fungicides, or other xenobiotics is critical to the protection and maintenance of ecosystem stability. Pesticides may accumulate in fish, producing adverse effects related to a number of mechanisms, such as endocrine disruption, neurotoxicity, and others; in addition, the adverse effect may be indirect, since fish is the last level of the trophic chain, and thus pesticides affecting the previous levels may have consequences on fish, such as in the case of herbicides affecting algae [16,17,18,19,20,21]. In this regard, environmental risk analysis plays a key role in adequately assessing possible xenobiotic side effects and, therefore, maintaining the ecological balance [22,23,24].

Since ecology is essentially a changing fragment of universal human reality, researchers need to measure their own research activities and observations against the problems, taking advantage of the achievements of specialists working in related and even distant areas [9,10]. The information received by the researcher must be convenient. The convenience of the information is linked to its heuristic value. In other words, the user (researcher or assessor) must not just receive some numerical values, but also the ability to perceive the phenomenon being studied (the endpoint) in a complex manner, considering the relationship with other phenomena.

The toxicity of organic compounds for fish is an important ecological indicator. It can be used to identify reliable substances and predict safer alternatives for agricultural applications. Thus, the information on the aquatic toxicity of organic compounds can be useful not only for the assessment of existing chemicals but also for the implementation of appropriate strategies for bringing safer chemicals to market. Obviously, some kind of information flow should be discussed here, since the list of compounds expected to be future pesticides is growing rapidly. Such an evolution should be considered to increase the efficacy of efforts using the existing list of substances aimed at moving toward greener pesticides. Indeed, there is a need to develop tools for modeling the ecological, biochemical, and physicochemical behavior of generally arbitrary substances that can find practical applications, such as pesticides, biocides, or substances in other sectors [25,26,27]. While there are guidelines to conduct experimental studies on adult fish and related endpoints, established by the Organization for Economic Co-operation and Development (OECD), the same agency encourages the development of quantitative structure–activity relationships (QSAR) or computer-aided risk assessment of different compounds as potential toxic agents for fish [28,29,30,31]. Naturally, this leads to the development of new and diverse computational approaches to solve the problems of modeling the toxicity of various compounds for fish [32,33,34]. Ideally, these new models should be related to and benefit from established scientific domains. Indeed, environmental toxicology is complex and multidisciplinary. It must incorporate physical, chemical, and biological understanding to resolve present and future environmental problems caused by toxic chemicals [35].

The aim of this work is to evaluate the effectiveness of new criteria for predictive potential simulation of the acute toxicity of organic compounds used as pesticides for Rainbow Trout (*Oncorhynchus mykiss*). These kinds of substances represent the applicability domain of the model, which is further refined considering the presence of rare molecular fragments, as discussed below. From a methodological point of view, the basic idea is that the Monte Carlo method can be a tool for modeling various endpoints in general and specifically for modeling Rainbow Trout toxicity. Thus, it is possible that the statistical quality of models of toxicity for Rainbow Trout can be improved by using new criteria of predictive potential, applying the Monte Carlo approach and its recent developments, using the index ideality correlation, correlation intensity index, and coefficient of conformity of correlative prediction. In our case, the Monte Carlo method is applied within the CORAL software (https://www.insilico.eu/coral, accessed on 27 May 2025) to develop in silico models, which use SMILES as the method to represent the chemical structure, extracting relevant molecular features from this simple representation.

## 2. Materials and Methods

### 2.1. Data

Acute toxicity values (expressed as lethal concentration 50%—LC50) of 311 organic pesticides for Rainbow Trout (*Oncorhynchus mykiss*) were taken from the literature [36]. The endpoints were expressed as the negative logarithm of lethal concentration (mM/L). The data were obtained according to the OECD guideline [28]. This guideline indicates several fish species. We used data on Rainbow Trout because they were more abundant.

### 2.2. Simulation Scheme

The basic steps of building a model within the framework of the used method are as follows: (1) Splitting into active and passive training sets. (2) Optimizing the correlation weights of molecular features extracted from SMILES. (3) Building a regression model linking the descriptor calculated by the correlation weights with the endpoint under study (toxicity for Rainbow Trout). (4) Validating the predictive potential of the model.

**Figure 1:**
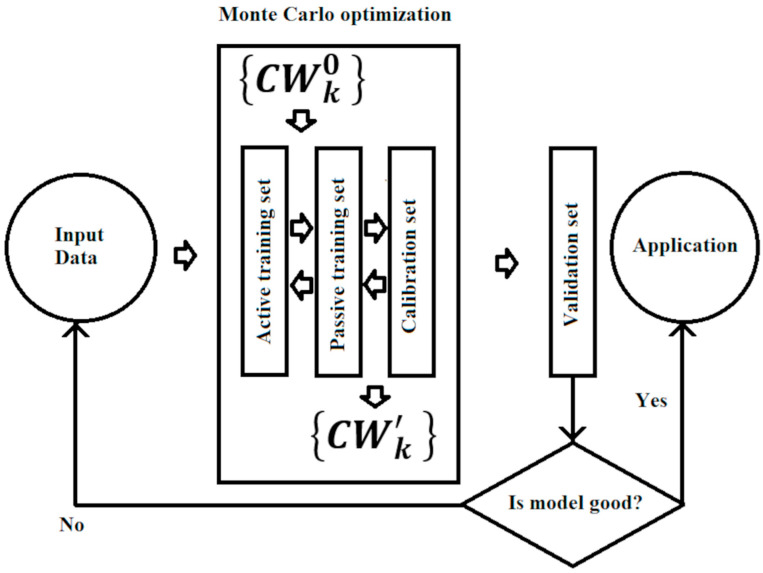
General scheme for building a model.

In practice, within step (1), the set of all compounds was randomly divided into four subsets of approximately the same number of substances: (i) an active training set (≈25%), (ii) a passive training set (≈25%), (iii) a calibration set (≈25%), and (iv) a validation set (≈25%). Within step (2), the active training set was used to build the model, and then the passive training set was used as an inspector of the model being built. Within step (3), the calibration set was used to determine the overall parameters of the model, avoiding overtraining. Finally, within step (4), the external validation set was used to evaluate the predictive potential of the resulting model (Figure 1). The same process was repeated five times to obtain a more robust statistical basis.

### 2.3. Optimal Descriptors

Modeling based on optimal descriptors involves several levels of modeling activities [37,38]. The first is the list of structural parameters of the molecules of the training set. The second level is based on the assigned threshold of the list of rare and the list of active parameters. The third level is the list of parameters that are apparent promoters of an increase or apparent promoters of a decrease in the endpoint.

The selected list of molecular features extracted from the dataset involves the so-called SMILES atoms, which are one symbol or a group of symbols that cannot be considered separately. In addition, pairs of atoms that are neighbors in the SMILES line are recruited in the simulation. Thus, in our modeling approach, the molecular features are directly extracted from the SMILES without using additional software to calculate complex molecular descriptors. Examples of these simple molecular features are presented below in Section 3.

The optimal descriptors considered here are calculated as follows:

(1) $$DCW\left(T,N\right)=\sum CW\left(S_{k}\right)+\sum CW\left({SS}_{k}\right)$$

CW(x) are correlation weights for molecular features extracted from SMILES. S_k_ is a SMILES atom; SS_k_ is a pair of SMILES atoms that are neighbors in the SMILES line. It should be noted that the inclusion of the above-listed features in the calculation scheme is more like conducting a public survey than strictly measuring “molecular individuality”. Such surveys often contain questions that are incorrect in several directions at once. For example, the question may be unclear to the respondent. In addition, the answers “Yes” or “No” may ambiguously relate to the goals of the survey. Nevertheless, sociological surveys are practiced, and often, the results of such surveys are ultimately useful.

### 2.4. Optimization of Correlation Weights

The correlation weights of SMILES attributes are calculated using CORAL software (https://www.insilico.eu/coral, accesed on 27 May 2025). The optimization process applied here involves special components termed index of ideality of correlation (IIC) [39] and correlation intensity index (CII) [40]. There is an analogy between the action of the above indices and the action of catalysts. However, if the catalyst should accelerate the chemical reaction, the IIC and CII should increase the “system’s attention” to that part of the training set whose position in the statistical probability aspect is closer to the status of the external validation set. In other words, the effect of these “catalysts” of the stochastic information process is aimed at improving the model for the calibration set. Unfortunately, this effect is useful for the calibration set, but, quite often, it reduces the performance observed on the initial (active and passive) training sets. As a result, the forecast is somewhat improved for the calibration set but worsens for the training sets. The influence of these factors can be regulated by using the weighting coefficients for the IIC and CII. The selection of these coefficients is carried out empirically—that is, based on the results of preliminary observations of the stochastic optimization system, with different weights for the IIC and CII. Having numerical data on the correlation weights, one can calculate the long-term toxicity under consideration with this equation:

(2) $$p{LC}_{50}=C_{0}+C_{1}\times DCW(T,N)$$

C_0_ and C_1_ are regression coefficients; T is the threshold used to define rare and non-rare SMILES attributes. SMILES attribute is considered rare if its frequency in the active training set is less than T (rare attributes are not considered; their correlation weights are equal to zero). N is the number of epochs of Monte Carlo optimization. In this study, T = 5 and N = 15 obtained from the computational experiments within the modeling optimization.

To obtain the correlation weights necessary for calculating the descriptors by Equation (1), the following target functions were used, where IIC, CII, and the coefficient of conformism of correlation prediction (CCCP) [40] were considered:

(3) $${TF}_{0}=R_{AT}+R_{PT}-\left|R_{AT}-R_{PT}\right|\times 0.1$$

(4) $${TF}_{1}={TF}_{0}+IIC\times 0.3$$

(5) $${TF}_{2}={TF}_{0}+CII\times 0.3$$

(6) $${TF}_{3}={TF}_{0}+CCCP\times 0.3$$

R_AT_ and R_PT_ are correlation coefficients between observed and calculated values of pLC_50_ observed for the active training set and the passive training set, respectively.

### 2.5. Applicability Domain

The applicability domain for the described model, calculated with Equation (2), defines the so-called statistical defects of SMILES attributes. These defects can be calculated as follows [37,38]:

(7) $$d_{k}=\frac{\left|{P(A}_{k})-{P^{\prime }(A}_{k})\right|}{N\left(A_{k}\right)+N^{\prime }(A_{k})}+\frac{\left|{P(A}_{k})-{P^{\prime \prime }(A}_{k})\right|}{N\left(A_{k}\right)+N^{\prime \prime }(A_{k})}+\frac{\left|{P^{\prime }(A}_{k})-{P^{\prime \prime }(A}_{k})\right|}{N^{\prime }\left(A_{k}\right)+N^{\prime \prime }(A_{k})}$$

where P(A_k_), P′(A_k_) P″(A_k_) are the probability of A_k_ in the active training set, passive training set, and calibration set, respectively; N(A_k_), N′(A_k_), and N″(A_k_) are frequencies of A_k_ in the active training set, passive training set, and calibration set, respectively. The statistical SMILES-defects (D_j_) are calculated as

(8) $$D_{j}=\sum_{k=1}^{NA}d_{k}$$

where NA is the number of non-blocked SMILES attributes in the SMILES.

A SMILES falls in the domain of applicability if

(9) $$D_{J}<2\times \bar{D}$$

### 2.6. Mechanistic Interpretation

The developed QSAR models allow for mechanical interpretation of the studied phenomena. With numerical data on the correlation weights of features that take place in several runs of the Monte Carlo optimization, one can extract three categories of these features [39,40]: (1) features that have a positive value of the correlation weight in all runs. These are promoters of endpoint increase; (2) features that have a negative value of the correlation weight in all runs. These are promoters of endpoint decrease; (3) features that have both negative and positive values of the correlation weight in different runs of the optimization. These are features with unclear roles (one cannot classify these features as promoters of an increase or decrease for the endpoint).

## 3. Results

Table 1 contains the statistical characteristics of models obtained with target functions TF_1_, TF_2_, and TF_3_. Table 1 shows the complete results obtained with the different subsets. The relevant results are those observed on the calibration set, which corresponds to the final model, and, of course, the results on the validation set, which are substances not used in the model building phase.

The number of outliers according to the statistical defect dk for the considered splits is 6, 5, 15, 4, and 9 for splits 1–5, respectively.

Table 2 contains an example of a mechanistic interpretation for a model observed in TF_3_- optimization. One can see that aromaticity, double bonds, chlorine, and sulfur atoms are promoters of an increase in pLC50 for Rainbow Trout, whereas nitrogen and oxygen atoms are promoters of a decrease in toxicity for Rainbow Trout. This is observed for this dataset. However, additional computational experiments are needed to assess how true these data will be for other compounds (which are absent in considered the dataset).

Table 3 contains the statistical characteristics of models for toxicity for Rainbow Trout suggested in the literature. A comparison of the results observed here in the case of the TF_3_ optimization for split 1 confirms that the statistical characteristics of the model obtained here are comparable or even better than those from the literature.

## 4. Discussion

In Table 1, one can see that the best predictive potential of the model of acute toxicity for Rainbow Trout was observed in the case of the optimization with target function TF_3_. As we commented in the previous section, the relevant values are those related to the calibration and validation sets. Indeed, the results on the active and passive training sets correspond to the initial steps of the model building. The model’s initial steps can be attracted by some peculiar substances, forcing the predictions of these substances, which may be relatively unusual and rare. Thus, these preliminary models are quite local, and in most cases, they cannot extract general rules. The use of IIC, CII, or CCCP leads to the attention of molecular features inherent in the calibration set, which “correctly” poses the attention to general characteristics, assigning a lower statistical relevance to the rare substances. This may lead to the above-mentioned paradox: the system acts in such a way as to preserve the statistical quality of the model on the calibration set, even if this “spoils” the model for the training sets.

**Figure 2:**
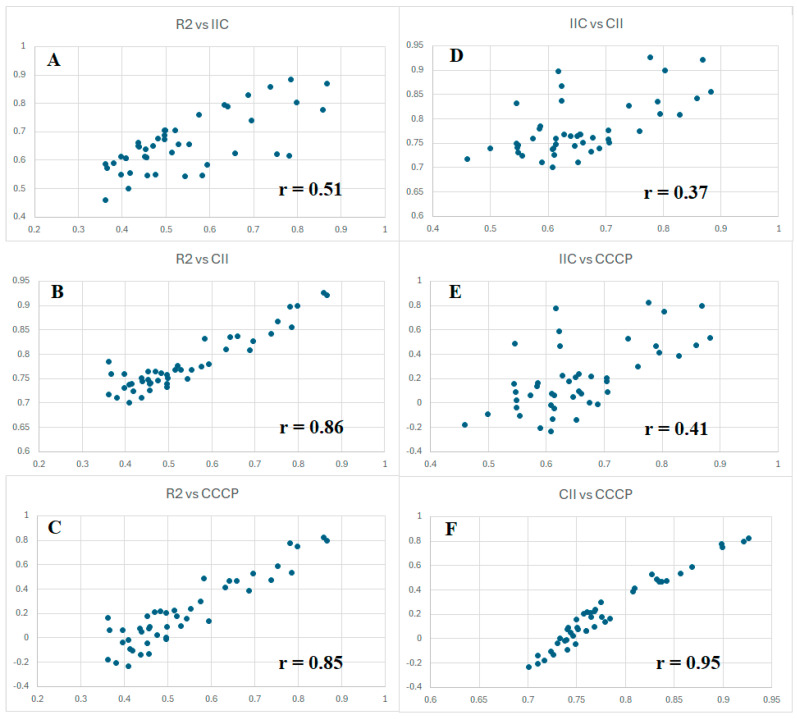
Comparison of the values of IIC, CII, and CCCP observed when developing toxicity models with three different target functions for five random splits into training and validation sets. The correlations of R^2^ and IIC (**A**); R^2^ and CII (**B**); R^2^ and CCCP (**C**); IIC and CII (**D**); IIC and CCCP (**E**); CII and CCCP (**F**) are presented.

However, since all three criteria of forecast potential considered here are aimed at the same goal, it seems reasonable to compare their values for all five splits, with three types of optimizations (target functions TF_1_, TF_2_, and TF_3_). Figure 2 graphically shows the proportionality of these values. Considering the results obtained with IIC (Figure 2A), high improvements were obtained using CII (Figure 2B) and CCCP (Figure 2C). It should be noted, nevertheless, that the observed situation is inherent in the conditions considered. Perhaps for other endpoints, this proportionality will have a different form.

Chronologically, the first criterion of predictive potential was tested using IIC. As can be seen in Figure 2, it weakly correlates with the value of the coefficient of determination (Figure 2A). However, the most successful in this study as a “catalyst of predictive potential”, CCCP, correlates with the value of the coefficient of determination much better (Figure 2C). It is important to notice, in the data presented in Table 1, that all three above criteria contribute to the improvement in predictive potential. There are correlations between the parameters that have been used. The most highly correlated values are CII and CCCP (Figure 2F). This is probably because these values have a similar origin. They are calculated based on the contributions of the correlation of opponents and supporters. However, CII is calculated based on the contributions of supporters only, while CCCP is calculated considering the contributions of both supporters and opponents of the correlation. Finally, CCCP has quite a good correlation with the determination coefficient (Figure 2C).

At present, the idea of information dynamics, which has some analogy with thermodynamics, is gaining more and more popularity [42]. The principles of infodynamics have been formulated, which, at first glance, are like the principles of thermodynamics. However, there is a certain inconsistency or even contradiction between the indicated ideologies. The first principle of thermodynamics can be formulated as “energy cannot be created or destroyed in an isolated system”. The first law of information dynamics can be formulated as “information cannot be created or destroyed but only transmitted or changed”. This principle is considered an all-encompassing one and therefore does not in any way imply the isolation of the system, unlike the first law of thermodynamics. The second law of thermodynamics states that the heat of a hotter system only flows toward a colder one. This is an irreversible process that always goes in the direction of increasing entropy (increasing chaos). In contrast to the second law of thermodynamics, the second law of information dynamics states that the information entropy (chaos) of systems containing information states must remain constant or decrease over time, reaching a certain minimum value at equilibrium.

According to the above discussion, it can be concluded that information systems exist similarly to thermal systems. In particular, the “technical” and “statistical” state of the model on the training set and those on the external validation set can be qualified as information systems. One would like to assume that the predictive ability of the model on the training set is a measure of the information content or information value, which can be indirectly, through the corresponding equations, extended to external information systems, i.e., to the validation set. Experience shows, however, that the value or reliability of the model on the validation set is rarely transferred from the training set without losses. One can say that the model is a message generated based on the analysis of the training set. Information entropy in this case can be defined as a measure of the uncertainty of the message source.

Despite the optimism of the second law of infodynamics, one cannot help but notice that in relation to research activities, information entropy (chaos) is far from a rare phenomenon. The desire for tools convenient for practical use often turns out to be futile, and Internet users, instead of the expected comfort, encounter a series of problems of both a bureaucratic and informational nature. If mass and energy can exist outside of human perception, then the information that one would like to talk about must be for human perception. From this perspective, it would be interesting to understand whether and, if so, how the ideas of infodynamics can be useful for QSAR analysis.

Information convenient for perception should be structured according to the user’s needs. This may be contrary to the second law of infodynamics, but structured information should not be complex, i.e., requiring many steps for practical application (understanding). An increase in the number of steps is clearly capable of increasing information entropy (chaos). However, the new ideology proposed in the field of any research work should be considered and assessed in terms of its prospects for solving research problems. Whether the laws of infodynamics will help in solving the problems of the QSAR can be shown by relevant experiments and observations.

Here, we consider a system of optimal descriptors extracted from SMILES [39,40]. This is a quite convenient approach to use databases where molecules are presented in a very compact form, containing as much information as possible, which can be sorted and, if necessary, shortened. They reduce the time of computer calculation while still allowing enough chemical representation. The representation of molecular structure can be performed in many ways. The most common ways to accomplish this for QSAR analysis are SMILES and InChI. However, InChI is designed to be used “behind the scenes” by computers [43]. It is usually derived from structure representations by software, whereas SMILES conveniently supports communication between humans and molecular architecture.

In philosophy, the concept of a “dialectical pair” is known, implying a pair of concepts capable of directing thought in some rationally constructive direction, for the formation of useful hypotheses for research work. Examples of dialectical pairs, often mentioned in the general philosophical context, are essence and phenomenon, quality and quantity, space and time, cause and effect, necessity and chance, reality and possibility, matter and consciousness, and information and uncertainty. The practice of working with optimal descriptors leads to the formulation of the following dialectical pair: randomness and prediction [44].

It is obvious that the stochastic processes considered are the personification of randomness. However, due to the objective functions used in the Monte Carlo optimization processes considered here, randomness becomes capable of creating a prediction based on the information provided by SMILES. This gives rise to a number of concerns related to the practice of using SMILES. Firstly, the SMILES used must be homogeneous in their origin, i.e., in the algorithms of their creation. Second, how informative are the selected SMILES? In other words, does the selected SMILES version provide adequate molecular structure information sufficient to identify the molecular features needed for a given endpoint? The structures can be aromatic, with 3D architecture, chirality, and other features. This issue is practically resolved based on the rule: all SMILES participating in the construction of the model must be constructed by the same algorithm. The same applies to SMILES included in the external verification.

Following the principle of “QSPR/QSAR are random events” [39,40], any conclusions related to QSPR/QSAR analysis must be confirmed based on the observation of not one but several distributions of training and validation samples.

Thus, the use of the SMILES format is convenient and adequate for modeling purposes, provided that the same algorithm to generate SMILES is used. Since more and more databases contain the SMILES structure, its use is facilitated. An advantage of our approach is that there is no need to calculate molecular descriptors, since the software we used only needs the SMILES, and the molecular features used for modeling purposes and components, which are alphanumerical symbols derived from the SMILES, as shown in Table 2. This largely simplifies the modeling process and, at the same time, provides an easy representation of the molecular components that may be associated with the adverse effect. Examples of molecular components associated with adverse effects are the presence of chlorine, sulfur, benzene rings, and molecular branching. Indeed, these SMILES attributes extracted by the software have a positive coefficient (CWs), which is consistent in all the cases. Conversely, other SMILES attributes have a negative coefficient, which means that substances containing these components are relatively less toxic. Examples of these mitigating factors are the presence of oxygen, nitrogen, or oxygen in a molecule with branching and the presence of aromatic rings with nitrogen in the ring. Thus, this information may represent useful suggestions when planning safer pesticides. However, it is obvious that SMILES is limited both by the ability to convey fine details of the molecular structure (for example, the possibility of rotational isomerism in dynamics) and complex interactions with the organisms. As we have seen above, Table 2 contains quite general components, without entering into more complex features. In addition, stochastic algorithms cannot be highly accurate. Therefore, the approach under consideration has limitations in accuracy and depends on the selected SMILES variety.

Still, we notice that the approach presented here is highly valuable. Table 3, which compares the results presented here with those obtained with other in silico models, clearly shows that the models presented here for toxicity for Rainbow Trout are among the best, in particular considering the values when predicting new substances: our model gave an R^2^ value of 0.89 on the validation sets, higher than those from the other models. The comparison with the values on the training set in Table 3, with all the substances, may be misleading, as discussed above, since it includes the values obtained in the initial phases of the model preparation using the active and training set; it is more correct to compare the results on the calibration set, as shown in Table 1: values range from 0.70 to 0.87 for the calibration set. Thus, the method presented here is convenient for a quick preliminary assessment of acute toxicity for Rainbow Trout (*Oncorhynchus mykiss*). Supplementary Materials section contains technical details on the model for split-1.

The study reported here is aligned with the efforts to use New Approach Methodologies (NAMs). NAMs cover a series of approaches, such as omics and in vitro methods, and include in silico models. We notice that the European Food Safety Authority (EFSA), which in Europe is dealing with pesticides, is exploring the use of NAMs [45]. This perspective aims to increase the sources of information to be used for hazard (in our case) and risk assessment, in general terms. Thus, it is consistent with the efforts to reduce the use of animal studies for ethical reasons, costs, and resources for laboratory in vivo experiments. It also introduces a positive vision, adding novel insights into toxicology. The complexity of the toxicological processes requires multiple tools, and thus initiatives in this direction are welcome. Regulatory considerations complement the scientific point of view. We mentioned that EFSA is actively moving in this direction. The different jurisdictions have different premises and contexts. For instance, in vivo studies are not allowed in Europe for cosmetics, and there is a trend to follow this in other countries. There is an intense dialogue between the scientific community and the authorities regarding a sound assessment of the hazard and risk properties of substances, and this depends on the different sectors, since different conditions apply [46]. Thus, our study is relevant to pesticides, as declared, but it is useful for other cases, too. Biocides are the closest case, since there is a large overlap between these two kinds of substances. Moreover, there are commonalities between some pesticides and some pharmaceuticals. For instance, antimycotic substances are used both as pesticides and pharmaceuticals. Furthermore, from a general point of view, pesticides as pharmaceuticals have a quite complex structure and have been developed to be active through different modes of action, and for this reason, it is possible that our model for pesticides may be applicable, at least partially, to pharmaceuticals.

## 5. Conclusions

Three criteria of predictive potential for models for acute fish toxicity of Rainbow Trout (*Oncorhynchus mykiss*) are presented here. The models used the Monte Carlo optimization of correlation weights of SMILES attributes. The best results were observed when using the CCCP (coefficient of conformism of correlation prediction), which gave statistical values similar or better than those obtained with other models for the same endpoint. The reliability and predictive potential of the approach considered is tested with five splits of the available data into training samples (active and passive), calibration sets, and external validation sets. The approach used is quite simple, since it only needs the SMILES structure, without the calculation of molecular descriptors. Information on the role of certain molecular features is provided by the model, increasing or decreasing the adverse effects. This information can be applied in the planning of safer pesticides. These models, as with all statistical models, have the limitation of being related to the information present in the training set. The meaning of these models is applicable to the general case represented by the population of substances in the training set and may fail if a quite different molecular skeleton is addressed. Improvements can be achieved using additional experimental data on new substances. It is suggested that the QSAR analysis can be enriched by the ideology of infodynamics if information is understood as something related to human perception. These models will be implemented on the VEGAHUB website (www.vegahub.eu, accessed on 27 May 2025) for free use.

## Figures and Tables

**Table 1:** The statistical quality of models obtained with target functions TF_1_, TF_2_, and TF_3_.

						TF_1_					
Split	Set *	n	R^2^	CCC	IIC	CII	Q^2^	CCCP	RMSE	F	N_A_
1	A	79	0.476	0.645	0.549	0.746	0.443	0.024	1.18	70	
	P	77	0.544	0.652	0.545	0.750	0.524	0.159	1.32	89	
	C	78	0.576	0.748	0.759	0.775	0.541	0.301	0.76	103	
	V	77	0.844	-	-	-	-	-	0.66	-	61
2	A	78	0.409	0.581	0.608	0.738	0.377	−0.019	1.28	53	
	P	78	0.409	0.542	0.608	0.701	0.377	−0.235	1.43	53	
	C	77	0.687	0.826	0.829	0.808	0.664	0.386	0.52	165	
	V	78	0.805	-	-	-	-	-	0.57	-	60
3	A	78	0.419	0.590	0.555	0.723	0.388	−0.107	1.41	55	
	P	77	0.381	0.543	0.589	0.710	0.348	−0.209	1.32	46	
	C	78	0.785	0.874	0.883	0.856	0.772	0.536	0.47	277	
	V	78	0.837	-	-	-	-	-	0.57	-	58
4	A	79	0.361	0.531	0.586	0.784	0.328	0.164	1.33	44	
	P	77	0.362	0.493	0.460	0.717	0.325	−0.182	1.49	43	
	C	78	0.738	0.839	0.859	0.842	0.715	0.474	0.53	214	
	V	77	0.831	-	-	-	-	-	0.51	-	61
5	A	78	0.453	0.624	0.640	0.765	0.425	0.177	1.37	63	
	P	78	0.414	0.615	0.499	0.740	0.384	−0.092	1.29	54	
	C	78	0.633	0.792	0.795	0.809	0.609	0.413	0.56	131	
	V	77	0.827	-	-	-	-	-	0.57	-	62
						**TF_2_**					
1	A	79	0.554	0.713	0.656	0.769	0.527	0.240	1.09	96	
	P	77	0.594	0.678	0.585	0.779	0.576	0.140	1.30	110	
	C	78	0.366	0.576	0.573	0.760	0.310	0.063	1.08	44	
	V	77	0.632	-	-	-	-	-	1.10	-	61
2	A	78	0.498	0.665	0.706	0.751	0.469	0.090	1.18	75	
	P	78	0.497	0.640	0.689	0.740	0.473	−0.014	1.32	75	
	C	77	0.659	0.800	0.623	0.837	0.636	0.466	0.62	145	
	V	78	0.810	-	-	-	-	-	0.70	-	60
3	A	78	0.497	0.664	0.705	0.757	0.472	0.203	1.31	75	
	P	77	0.497	0.623	0.675	0.733	0.467	0.005	1.20	74	
	C	78	0.753	0.837	0.623	0.868	0.733	0.591	0.57	232	
	V	78	0.826	-	-	-	-	-	0.64	-	58
4	A	79	0.482	0.651	0.677	0.761	0.4537	0.218	1.19	72	
	P	77	0.515	0.656	0.628	0.768	0.4897	0.223	1.28	79	
	C	78	0.642	0.801	0.790	0.835	0.6105	0.465	0.65	136	
	V	77	0.720	-	-	-	-	-	0.78	-	61
5	A	78	0.529	0.692	0.657	0.768	0.505	0.097	1.27	85	
	P	78	0.521	0.706	0.704	0.776	0.496	0.179	1.21	83	
	C	78	0.583	0.742	0.545	0.832	0.543	0.484	0.66	106	
	V	77	0.778	-	-	-	-	-	0.72	-	62
						**TF_3_**					
1	A	79	0.440	0.611	0.646	0.744	0.406	0.052	1.22	60	
	P	77	0.458	0.629	0.547	0.741	0.429	0.093	1.41	63	
	C	78	0.696	0.823	0.741	0.827	0.675	0.530	0.64	174	
	V	77	0.886	-	-	-	-	-	0.62	-	61
2	A	78	0.437	0.608	0.661	0.752	0.405	0.079	1.25	59	
	P	78	0.437	0.586	0.651	0.710	0.405	−0.140	1.40	59	
	C	77	0.799	0.890	0.803	0.899	0.785	0.749	0.44	297	
	V	78	0.886	-	-	-	-	-	0.47	-	60
3	A	78	0.456	0.627	0.610	0.740	0.429	0.080	1.36	64	
	P	77	0.456	0.567	0.611	0.726	0.425	−0.134	1.25	63	
	C	78	0.867	0.919	0.869	0.921	0.858	0.796	0.37	495	
	V	78	0.887	-	-	-	-	-	0.46	-	58
4	A	79	0.397	0.568	0.614	0.759	0.368	0.064	1.29	51	
	P	77	0.397	0.544	0.549	0.730	0.362	−0.039	1.43	49	
	C	78	0.858	0.906	0.777	0.926	0.847	0.827	0.40	459	
	V	77	0.883	-	-	-	-	-	0.42	-	61
5	A	78	0.470	0.639	0.651	0.764	0.442	0.208	1.35	67	
	P	78	0.453	0.652	0.614	0.749	0.424	−0.043	1.27	63	
	C	78	0.781	0.872	0.617	0.898	0.765	0.776	0.43	271	
	V	77	0.881	-	-	-	-	-	0.48	-	62

*A = active training set; P = passive training set; C = calibration set; V = validation set; R^2^ = determination coefficient; CCC = concordance correlation coefficient; IIC = index of ideality of correlation; CII = correlation intensity index; Q^2^ = cross-validated R^2^; RMSE = root mean squared error; MAE = mean absolute error; F = Fischer F-ratio; N_A_ = the number of active (non-rare) SMILES attributes.

**Table 2:** Mechanistic interpretation for the model based on TF_3_ optimization with split 1.

No.	SMILES Attributes	CWs Probe 1	CWs Probe 2	CWs Probe 3	NA	NP	NC	d_k_
1	1...........	0.353	1.271	0.414	70	65	61	0.0011
2	=...........	0.460	0.181	0.482	62	65	62	0.0006
3	c...1.......	0.823	0.266	0.290	57	44	56	0.0019
4	c...c.......	0.484	0.355	0.005	55	44	50	0.0017
5	(...(.......	0.904	0.393	0.512	39	33	36	0.0012
6	Cl..........	1.433	0.096	0.788	38	30	32	0.0018
7	S...........	1.643	1.467	1.151	22	28	21	0.0027
1	O...(.......	−0.128	−0.600	−0.306	59	55	56	0.0004
2	N...........	−0.885	−0.481	−0.966	51	39	45	0.0021
3	N...(.......	−0.685	−0.089	−0.094	37	28	33	0.0021
4	C...=.......	−0.243	−0.087	−0.460	31	37	28	0.0025
5	1...(.......	−0.506	−0.122	−0.522	29	24	24	0.0015
6	n...........	−0.063	−0.402	−0.277	26	13	21	0.0053
7	n...c.......	−0.807	−0.276	−0.770	24	11	21	0.0057

**Table 3:** Comparison of models of toxicity for Rainbow Trout suggested in the literature.

The Number of Compounds in the Training Set	Determination Coefficient for Training Set	The Number of Compounds in Validation Set	Determination Coefficient for Validation Set	Reference
249	0.80	62	0.81	[36]
233	0.67	78	0.86	[39]
66	0.80	22	0.74	[41]
234	0.53	77	0.89	In this work

## Data Availability

The original contributions presented in this study are included in the article/Supplementary Material. Further inquiries can be directed to the corresponding author.

## Supplementary Materials

The following supporting information can be downloaded at: https://www.mdpi.com/article/10.3390/jox15030082/s1, Table S1: Technical details for split 1.
